# Fluorometric Liposome Screen for Inhibitors of a Physiologically Important Bacterial Ion Channel

**DOI:** 10.3389/fmicb.2021.603700

**Published:** 2021-03-01

**Authors:** Andreia S. Fernandes, António Pombinho, Celso M. Teixeira-Duarte, João H. Morais-Cabral, Carol A. Harley

**Affiliations:** ^1^Instituto de Investigação e Inovação em Saúde (i3S), Universidade do Porto, Porto, Portugal; ^2^Instituto de Biologia Molecular e Celular (IBMC), Universidade do Porto, Porto, Portugal; ^3^Programa Doutoral em Biologia Molecular e Celular (MCbiology), Instituto de Ciências Biomédicas Abel Salazar (ICBAS), Universidade do Porto, Porto, Portugal

**Keywords:** KtrAB, high-throughput screen, antibacterial target, K^+^ homeostasis, *Bacillus subtilis*, superfamily of potassium transporters, membrane protein

## Abstract

The bacterial K^+^ homeostasis machinery is widely conserved across bacterial species, and different from that in animals. Dysfunction in components of the machinery has an impact on intracellular turgor, membrane potential, adaptation to changes in both extracellular pH and osmolarity, and in virulence. Using a fluorescence-based liposome flux assay, we have performed a high-throughput screen to identify novel inhibitors of the KtrAB ion channel complex from *Bacillus subtilis*, a component of the K^+^ homeostasis machinery that is also present in many bacterial pathogens. The screen identified 41 compounds that inhibited K^+^ flux and that clustered into eight chemical groups. Many of the identified inhibitors were found to target KtrAB with an *in vitro* potency in the low μM range. We investigated the mechanisms of inhibition and found that most molecules affected either the membrane component of the channel, KtrB alone or the full KtrAB complex without a preference for the functional conformation of the channel, thus broadening their inhibitory action. A urea derivative molecule that inhibited the membrane component of KtrAB affected cell viability in conditions in which KtrAB activity is essential. With this proof-of-concept study, we demonstrate that targeting components of the K^+^ homeostasis machinery has the potential as a new antibacterial strategy and that the fluorescence-based flux assay is a robust tool for screening chemical libraries.

## Introduction

Potassium is the most abundant inorganic cation in cells from all organisms. In bacteria, K^+^ homeostasis is related to intracellular turgor and membrane potential, and is involved in the mechanisms of adaptation to environmental stress such as changes in extracellular pH or osmolarity (reviewed by Epstein, 2003). For example, during adaptation to hyper-osmotic shock the concentration of K^+^ rises from ~300 to 600–700 mM (Richey et al., 1987; Whatmore et al., 1990).

The K^+^ homeostasis machinery in bacteria includes only a few types or families of K^+^ importers and exporters. An important family of bacterial K^+^ importers is the superfamily of potassium transporters (SKT), which are fast ion importers present in all kingdoms of life except animals. This superfamily includes the subgroup of prokaryotic TrK/Ktr channels, which show low selectivity for K^+^ (Cao et al., 2013; Levin and Zhou, 2014; Diskowski et al., 2015). SKT members evolved from a simpler bacterial KcsA-like channel, with which they share the core structural motif of transmembrane helix-pore-transmembrane helix (TM-PLoop-TM), with the pore segment including a selectivity filter (Durell and Guy, 1999; Durell et al., 1999; Corratge-Faillie et al., 2010). The ion conducting membrane part of prokaryotic TrK/Ktr channels, TrKH/KtrB, is formed by a dimer of four TM-PLoop-TM repeats assembled around a central pore. Crystal and cryo-EM structures of Trk from *Vibrio parahaemolyticus* and Ktr complexes from *Vibrio alginolyticus* and *Bacillus subtilis* show a dimeric membrane protein assembled with a cytoplasmic tetrameric or octameric gating ring composed by the regulators of conductance of K^+^ (RCK) domains TrkA and KtrA, respectively (Cao et al., 2013; Vieira-Pires et al., 2013; Diskowski et al., 2017). TrkA and KtrA are nucleotide-dependent RCK domains regulated by ATP and ADP. Binding of the nucleotides induces conformational changes in the gating ring that propagate to the transmembrane subunit and lead to an active (ATP-bound) or inactive (ADP-bound) channel (reviewed by Schrecker et al., 2019). In some bacteria and archaea, cyclic-di-AMP also binds to these RCK domains and is proposed to inhibit K^+^ flux (Corrigan et al., 2013; Bai et al., 2014; Kim et al., 2015; Commichau et al., 2018, 2019; Rocha et al., 2019). In addition it has been demonstrated that cyclic-di-AMP binds to the *ydaO* riboswitch in the *ktrAB* operon of *B. subtilis* inhibiting transcription and therefore regulating protein levels in the cell (Nelson et al., 2013; Gundlach et al., 2017).

Members of the TrK/Ktr subfamily of SKT have been implicated in resistance to antibiotics. Screening of a library of insertion mutations in *Mycobacterium smegmatis* revealed mutations in TrkA that resulted in changes to the proton motive force (PMF) and reduced bacterial susceptibility to antibiotic challenge (Castaneda-Garcia et al., 2011). *Escherichia coli* strains adapted to various aminoglycoside antibiotics revealed mutations in TrkH. It was suggested that resistance to the aminoglycosides resulted from an altered function of TrkH that affected the PMF (Lazar et al., 2013; Oz et al., 2014). Another TrkH point mutation was identified in a highly streptomycin-resistant *Salmonella enterica* strain (Wistrand-Yuen et al., 2018). In addition, a point mutation in TrkH was identified in a *Streptococcus pneumonia* pneumococcal isolate resistant to non-aminoglycoside antibiotics (Pan et al., 2018), and KtrA was found to confer resistance to aminoglycosides and fitness advantage during infection in *Staphylococcus aureus* (Gries et al., 2013).

Strikingly, TrK/Ktr proteins have also been implicated in virulence of pathogenic bacteria. In a *Campilobacter jejuni* transposon insertion screening, KtrA and KtrB were identified as virulence factors for mouse intestinal colonization. The role of KtrA in virulence was confirmed by observing that its knockout mutant was impaired in colonization, and reintroduction of the gene restores colonization capacity (Gao et al., 2017). TrK transposon mutants arose in two different screenings for virulence factors on the pathogenic bacteria *Aeromonas hydrophila* and *Pectobacterium wasabie*, and for both their role in virulence was confirmed (Valente and Xavier, 2016; Pang et al., 2017). In addition, inactivation of TrK in *Mycobacterium tuberculosis* leads to attenuation of host colonization (MacGilvary et al., 2019), and deletion of TrkA in *S. enterica* leads to attenuation of cell invasion and animal infection, which further increases with additional deletion of the other K^+^ importers Kup and Kdp (Liu et al., 2013). The function of K^+^ importers in virulence probably results from their role in adaptation mechanisms that are crucial for the ability of the pathogen to survive in different environments in the host.

In this study, we explore the viability of screening for novel inhibitors of the SKT proteins by using KtrAB from *B. subtilis* as a model channel. This is an extensively studied protein, with its structural and functional properties well characterized, both *in vitro* and *in vivo* (Holtmann et al., 2003; Albright et al., 2006; Vieira-Pires et al., 2013; Szollosi et al., 2016; Gundlach et al., 2017; Teixeira-Duarte et al., 2019). We have adapted an established fluorescence-based liposome flux assay (Su et al., 2016; Teixeira-Duarte et al., 2019) to monitor K^+^ efflux through KtrAB into a 96-well plate format and executed a proof-of-concept screening campaign in which we screened a diverse library with 10,000 compounds. In addition, we have established a secondary lead follow-up screening strategy in which we have identified a urea-based derivative that has a specific effect on channel activity.

## Materials and Methods

### Cloning, Protein Expression, Purification, and Incorporation Into Liposomes

*Bacillus subtilis* KtrAB, KtrAB with R417K mutation in KtrB, and KtrB proteins were expressed, purified, and incorporated into liposomes as previously described (Teixeira-Duarte et al., 2019), with the following modifications: EDTA was absent from KtrA purification, KtrB expression was induced for 3 h, KCl and EDTA were absent from the KtrB lysis buffer, and then liposomes were subjected to ultracentrifugation at 267,000 × *g* for 25 min at 4°C. The liposomes were resuspended in half the initial volume for concentration before flash freezing in liquid nitrogen.

*Bacillus cereus NaK* DNA was a kind gift of Y. Jiang. The N-terminally truncated *NaK_ΔN19_* was cloned into pQE60 by Gibson Assembly (Gibson et al., 2009) with appropriate primers (TAAGCTTAATTAGCTGAGCTTGGACTCC and GTTAATTTCTCCTCTTTAATGAATTCTGTGTGAAATTGTTATC to amplify pQE60, and AATTCATTAAAGAGGAGAAATTAACATGTGGAAAGATAAAGAATTTCAAGTATTATTTGTATTAACAATTTTGAC and GATCTATCAACAGGAGTCCAAGCTCAGCTAATTAAGCTTATTAGTGATGGTGATGGTGATGAGATCTG to amplify *NaK_∆N19_*), and the F92A mutation was introduced by site-directed mutagenesis with primers TTATTGGGATTGGACTAGTGGCTGGATTTATTCATAAGTTAGCAG and CTGCTAACTTATGAATAAATCCAGCCACTAGTCCAATCCC. NaK_ΔN19/F92A_ protein was expressed and purified as described (Alam and Jiang, 2009). Incorporation of *B. cereus* NaK_ΔN19/F92A_ protein into liposomes was performed as for *B. subtilis* KtrAB.

### K^+^ Flux Fluorescence-Based 96-Well Assay and Compound Screen

A screening campaign was conducted using a Chembridge DiverSet library (Chembridge, San Diego, CA, United States) consisting of 10,000 compounds chosen for their structure diversity. Compounds were stored at −20°C in 1 mM in DMSO in a 384-well format.

The K^+^ flux fluorescence-based screening assay was adapted from Su et al. (2016) and Teixeira-Duarte et al. (2019). It is based on the quenching of the pH gradient-sensitive dye 9-amino-6-chloro-2-methoxyacridine (ACMA) due to a proton influx that is coupled to the K^+^ efflux through KtrAB. Proteoliposomes in HEPES/NMG Buffer pH8.0 [10 mM HEPES, 7 mM NMG buffer (pH 8),150 mM KCl, 0.1 mM ATP, and 0.5 mM MgCl_2_] were 100-fold diluted in the same buffer containing 0.56 μM ACMA and 50 mM choline chloride instead of KCl. In this assay format, we used the lowest ACMA concentration to minimize potential compound interference. The mixture was added to 96-well black, flat-bottomed microplates (Greiner Bio-One, Monroe, NC, United States), 100 μl per well, and the plate was centrifuged at 1,000 rpm for 1 min in a bench top centrifuge. A Janus automated Workstation (PerkinElmer, Waltham, MA, United States) equipped with a pin tool (V&P Scientific, San Diego, CA, United States) was used to transfer 0.1 μl of compound from the 384-well library plates to the 96-well plates already containing the proteoliposome mixture such that the assay contained a final concentration of 1 μM compound and 0.1% DMSO. Wells in column 12 of each assay plate contained no compound and were used as negative controls representing no inhibition. The plates were transferred to a Synergy MX plate reader (BioTek, Winooski, VT, United States) and the fluorescence of ACMA was recorded over time at 490/20 nm with excitation 410/20 nm, for 2 min 40 s, using the maximum number of measurements per data point; in this way, we maximized read accuracy without excessively prolonging total assay time, which could compromise liposome stability. The assay was started with the addition of the protonophore carbonyl cyanide m-chlorophenyl hydrazine (CCCP) to a final concentration of 1 μM using a Multidrop Combi dispenser (Thermo Scientific, Waltham, MA, United States), and the fluorescence was measured every 12 s for 5 min, a time point at which quenching steady-state has been reached in the absence of inhibition (Teixeira-Duarte et al., 2019).

To measure K^+^ flux mediated by the KtrAB_R417K_-ADP complex ADP replaced ATP in the liposome preparation and in the assay buffer. To measure K^+^ flux from KtrB or NaK_*Δ*N19/F92A_, nucleotides were absent from the assay buffer, ACMA concentration was 1.12 μM, and the KtrB assay was run for 15 min.

### K^+^ Transport Fluorescence-Based Cuvette Assay

KtrB liposomes were prepared as described above except that they were not concentrated by ultracentrifugation. They were incubated with KtrA at a ratio 1:0.6 KtrB:KtrA (w:w) for at least 1 h at room temperature, diluted to a final volume of 2 ml in buffer containing ACMA, as above, plus 2 mM MgCl_2_ and 150 mM choline chloride. Measurements were done in polymethacrylate cuvettes (Merck, Germany) with a PTFE magnetic stirrer (*ϕ* 9 × 6 mm). Fluorescence at 480 nm, upon excitation at 410 nm, was recorded on a FluoroMax-4 (Horiba, Edison, NJ, United States) spectrofluorometer in 2 s intervals, with constant stirring. The assay was started by the addition of CCCP as above, and 300 nM valinomycin (Val) was added after 500 s to estimate the fraction of active liposomes.

### Screening Analysis, Statistics, and Retesting

Gen5 software (BioTek) was used to analyze all screening results. Two different types of analyses were performed: initial velocity and final fluorescence intensity. *Z*’ values were calculated within each assay plate and were found to be 0.1–0.7 (mean 0.4) for the initial velocities, and 0.7–0.95 (mean 0.9) for the final fluorescence intensities, indicating acceptable and excellent quality screens (Zhang et al., 1999), respectively. Fluorescence curves that gave changes of 2× SD from the initial velocity mean or 3× SD from the final fluorescence intensity mean were individually inspected. Clear false positives, generally due to compound fluorescence, were discarded, and the remaining compounds were retested at 1 μM in triplicate using samples from the original screening library plates and 12 negative controls wells per plate. Analysis for confirmation of these screening hits was done as for the initial screening, using the mean of the negative controls.

### Dose-Response

Compounds with confirmed activity were ordered from Chembridge, Hit2Lead and dose-response assays were performed as described above, in the presence of 50 (in plate format) or 150 mM (in cuvette) choline chloride, in triplicate, with compound concentrations ranging from 8 nM to 128 μM (depending on the compound), and at least 8 negative controls per plate. To test if compounds destabilized liposomes leading to false positive effects, empty liposomes were also assayed in the presence of high concentrations of compounds, by adding the K^+^ ionophore Val to a final concentration of 1 μM at the end of the assay. If, in the presence of a compound concentration, the decrease in fluorescence after Val addition was smaller than in the absence of compound, it was concluded that at this concentration, the compound destabilized liposomes, dissipating the K^+^ gradient, and it was removed from the dose-response analysis of proteoliposomes.

For data collected in plate format, dose-response curves were generated using final fluorescence intensities, by calculating the percentage of inhibition relative to the final fluorescence intensity before addition of CCCP.

For data collected in cuvette format, fluorescence curves were normalized using two different procedures: (1) using as maximum the initial fluorescence (before CCCP addition) and as minimum the fluorescence after Val addition and (2) using as maximum the initial fluorescence (before CCCP addition) and as minimum the fluorescence read immediately before Val addition. Initial velocities were determined from normalized curves as the slope of the straight line that fitted the first (at least five) time points in the curve, after addition of CCCP. Dose-response curves were generated using initial velocities, either by plotting the percentage of inhibition as a fractional change of maximum initial velocity, obtained in the absence of compound or by plotting the velocity value in function of compound concentration.

All dose-response curves were fitted with a Hill equation,

$$y=A+\left(B-A\right)\;\frac{x^{n_{H}}}{K_{I}{}^{n_{H}}+x^{n_{H}}},$$

in which A is the minimum inhibition or maximum velocity, B is the maximum inhibition or minimum velocity, *x* is the compound concentration, K_I_ is the constant of 50% inhibition, and n_H_ is the Hill coefficient.

### KtrA Thermal Shift Assay

Forty microliters of KtrA protein at a final concentration of 2.4 μM in HEPES/NMG buffer pH8.0 containing 5 mM DTT, and Sypro Orange Dye (Sigma-Aldrich, St. Louis, MO, United States) at a final concentration of 2.5×, were mixed with 10 μl of compound at a final concentration of 10 μM or 0.1% DMSO, as control. Assay was run in a 96-well white microplate (Bio-Rad, Hercules, CA, United States), in triplicate. After a centrifugation in a bench top centrifuge at 1,500 rpm for 1 min, plates were heated from 25 to 75°C in 0.5°C increments in an IQ5 Real Time Detection System (Bio-Rad) with a pause of 30 s at each temperature. Unfolding of KtrA was monitored using excitation and emission filters 545/30 nm and 585/20 nm, respectively.

### Growth Curves of *B. subtilis*

All *B. subtilis* cultures were performed in modified Spizizen’s minimal medium, containing 2 g/L (NH_4_)_2_SO_4_, 1 g/L sodium citrate, 0.2 g/L MgSO_4_·7H_2_O, 5 g/L glucose, 50 mg/L tryptophan, 50 mg/L phenylalanine, 125 mg/L MgCl_2_·6H_2_O, 7.3 mg/L CaCl_2_·2H_2_O, 13.5 mg/L FeCl_2_·6H_2_O, 1 mg/L MnCl_2_·4H_2_O, 1.7 mg/L ZnCl_2_, 0.43 mg/L CuCl_2_·2H_2_O, 0.3 mg/L CoCl_2_, and 0.6 mg/L NaMoO_4_·2H_2_O. Concentrations of phosphate salts (K_2_HPO_4_, KH_2_PO_4_, Na_2_HPO_4_, and NaH_2_PO_4_) were adjusted for the appropriate K^+^ concentration, keeping the total concentration of the monovalent cations (Na^+^ and K^+^) as 150 mM.

*Bacillus subtilis* JH642 (*pheA1 trpC2 citS642*; BGSCID 1A96) results from an 18-kb deletion on *B. subtilis* 168 (*trpC2*; BGSCID 1A1) genome (Srivatsan et al., 2008). *B. subtilis* 168 ∆ (*ktrB::kan*) strain, in which the ktrB gene is disrupted by a kanamycin resistance cassette, was constructed as follows. Genomic DNA of *B. subtilis* 168 ∆ (*ktrB::kan*) (obtained from the Bacillus Genetic Stock Center) was isolated with Gene JET Genomic DNA Purification kit (Thermo Scientific). The kanamycin resistance cassette flanked by KtrB sequences was amplified with primers GGCGGCAGCATTTGTAAAGAAC and CGCCTCTATTAAAGACAGTGCCGTTC. *B. subtilis* 168 was then transformed with the amplified DNA fragment according to Yasbin et al. (1975). To avoid suppressor mutations due to low K^+^ concentrations, positive transformants were selected in the presence of 10 g/L KCl, in addition to 7.5 μg/ml kanamycin.

All strains were grown in the presence of 1 mM K^+^ for 6 h at 37°C before being inoculated into 96-well sterile flat-bottomed microplates (Corning, Durham, NC, United States), at an initial OD_600_ 0.05, in a total volume of 100 μl supplemented with the appropriate concentration of compounds. Plates were covered with Breathe-Easy sealing tapes (Diversified Biotech, Dedham, MA, United States), which allow gas exchange, placed in a Synergy 2 or MX plate reader for 15 h at 37°C with medium agitation, and the OD_600_ was recorded every 30 min.

## Results

### Initial Screening and Retest Confirmation

**Figure 1:**
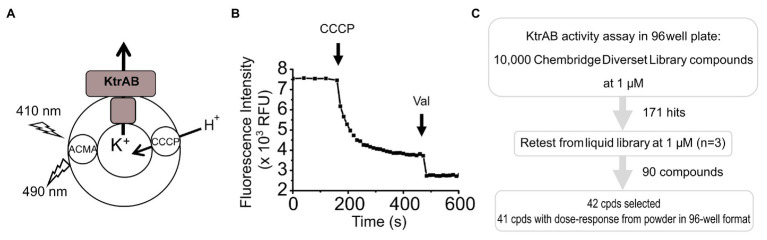
Screening assay. **(A)** Scheme of reconstituted KtrAB proteoliposomes in which quenching of 9-amino-6-chloro-2-methoxyacridine (ACMA) fluorescence is monitored in response to fluxes of K^+^ and H^+^ ions driven by KtrAB and CCCP. KtrAB is randomly oriented in liposomes and it is likely that more than one membrane protein complex are present in each liposome. **(B)** Example of a curve of ACMA fluorescence obtained from a 96-well plate format. Carbonyl cyanide m-chlorophenyl hydrazine (CCCP) and Val addition are represented by arrows. See text for a detailed explanation of the assay. **(C)** Scheme of the high throughput screening methodology followed in this study.

To identify regulators of K^+^ flux through the KtrAB channel, we screened 10,000 compounds at a final compound concentration of 1 μM from the Chembridge DiverSet library, using a modified fluorescence-based liposome flux assay (Su et al., 2016; Teixeira-Duarte et al., 2019) in a 96-well format (Figures 1A,B). In this assay, the ATP-bound KtrAB complex is reconstituted into liposomes in the presence of a high concentration of potassium (150 mM KCl). This reconstitution method allows both orientations of the complex, and both orientations are functional in the assay. The proteoliposomes are then diluted in a K^+^-free buffer containing ATP, creating a K^+^ gradient that drives K^+^ efflux through KtrAB. K^+^ efflux is counter balanced by H^+^ influx facilitated by the proton ionophore CCCP, creating a pH gradient that results in quenching of ACMA fluorescence. We followed quenching of ACMA over time after the addition of CCCP, and determined two parameters, the initial velocity and the fluorescence intensity at a final time-point, both of which reflect KtrAB activity.

To evaluate the non-specific impact of compounds on liposome integrity, we added the K^+^ ionophore Val at the end of the assay, which results in the total dissipation of the K^+^ gradient and rapid fluorescence quenching to a baseline level if liposomes are intact. In addition, quenching curves can be normalized to the total fluorescence quenching after Val addition. This ionophore was only included at the single dose retesting and dose-response steps of the screen.

In the initial screen, compounds were defined as “hits” if either the initial velocity diverged 2× SDs from the mean initial velocity in the absence of compound or the final fluorescence intensity diverged 3× SDs from the mean final fluorescence intensity in the absence of compound. This relatively wide cutoff was deliberately used so that we would not “miss” any potential hit compounds. Using these criteria, 171 compounds were identified resulting in an overall hit rate of 1.7% (Figure 1C; Supplementary Figure S1). The initial hits were retested in triplicate from liquid samples at 1 μM and 90 compounds retest-confirmed, resulting in a 52% retest confirmation rate.

### Dose-Response Confirmation

**Figure 2:**
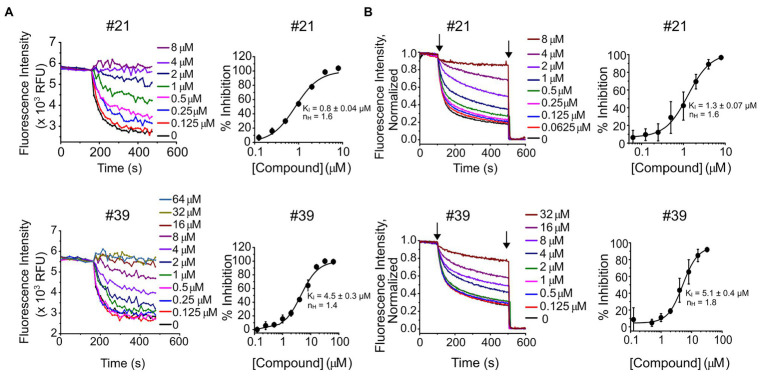
Dose-response of compounds #21 and #39. (**A**, left) – Representative experiments of K^+^ flux assays performed in 96-well plate with the indicated concentrations of compound #21 (top) or #39 (bottom). (**A**, right) – Percentage of inhibition of KtrAB activity determined using the final fluorescence from curves (on the left) as function of compound concentration, for compound #21 (top) or #39 (bottom). Mean ± standard deviation (SD) of triplicates are shown fitted with Hill equation and indicated n_H_ and K_I_ ± standard error (SE) parameters. See Supplementary Figure S3 for dose-response curves of all compounds. (**B**, left) – Representative experiments of K^+^ flux assays performed in cuvette with the indicated concentrations of compound #21 (top) or #39 (bottom). Curves were normalized using as maximum the initial fluorescence (before CCCP addition) and as minimum the fluorescence read after valinomycin (Val) addition. (**B**, right) – Percentage of inhibition of KtrAB activity determined using initial velocities determined from curves (on the left) as function of compound concentration, for compound #21 (top) or #39 (bottom). Mean ± SD of triplicates are shown fitted with Hill equation and indicated n_H_ and K_I_ ± SE parameters. Arrows indicate addition of CCCP (first) and Val (second).

Seventy of the 90 compounds that retest-confirmed were arranged into eight structural groups defined by heterocyclic ring backbone structure. Forty-two of these compounds were selected based on their structure and percentage of inhibition, and re-ordered as powders from Chembridge Hit2Lead for dose-response confirmation (Figure 1C). From those, 41 compounds showed a dose-response based on final fluorescence intensities and in 40 cases the dose-response could be fit with a Hill equation, deriving a constant of inhibition (K_I_) and Hill coefficient (n_H_) (Supplementary Table S1). In each case, we excluded from the dose-response curves the compound concentrations that had a deleterious effect on liposome integrity, observed when measuring the response of empty liposomes (without KtrAB) to Val in the presence of compound (Supplementary Figure S2; Supplementary Table S2). Representative dose-responses are shown in Figure 2A, for compounds #21 and #39 and Supplementary Figure S3 shows all dose responses. We further validated our plate-based assay format by repeating the dose-response of these compounds in a more rigorous single cuvette format. The cuvette data were analyzed using two approaches, by determining initial velocities from Val -normalized curves (Figure 2B) and by determining initial velocities from curves normalized to the final time-point on the segment after addition of CCCP only (Supplementary Figure S4). The K_I_ values determined varied less than 2 fold in magnitude and were consistent in both assay formats and analysis method used.

### Investigating the mechanism of compound inhibition

Many compounds showed a dose-response with K_I_ ~ 1 μM and were distributed throughout the different structural groups identified (Supplementary Table S1). Therefore, we decided to characterize in more detail nine representative compounds, outlined in Table 1 (compounds #21, #6, #31, #25, #3, #36, #39, #1, and #41), focusing on the following effects: binding site; state-dependent binding; binding specificity, and *in vivo* growth phenotype.

**Table 1:** Selection of representative compounds.

Compound #	Family	Structure	K_I_ (μM)	n_H_
21	Benzimidazols	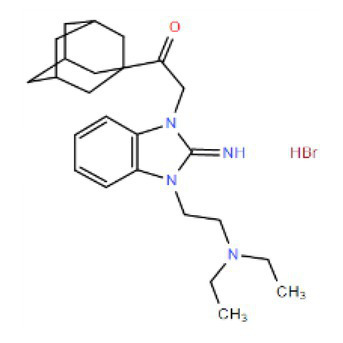	0.8	2
6	Piperazines	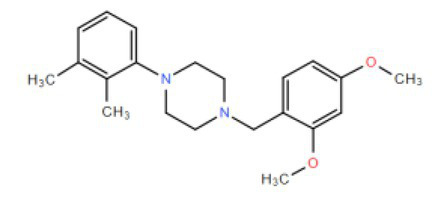	1	1.0
31	Piperidines	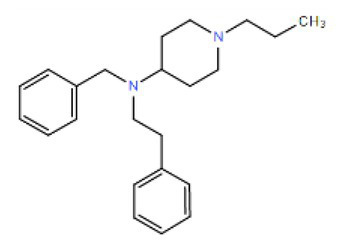	1	1.5
25	Phenoxybutyl piperidines and related	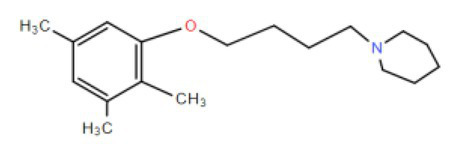	2	1.1
3	Azepanes	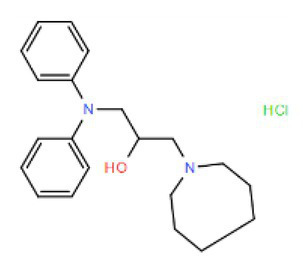	2	1.3
36	Ureas	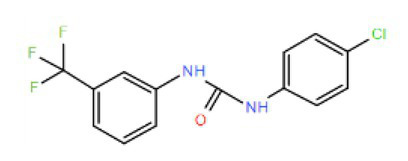	8	1.5
39	Phenylethylamines and related	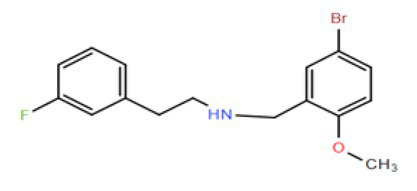	4	1.6
1	No family	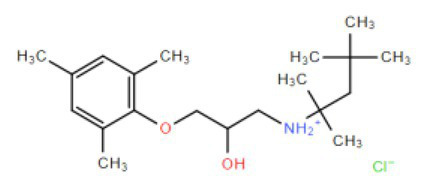	2	1.5
41	No family	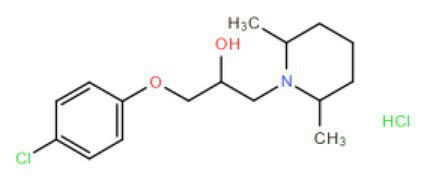	3	1.8

**Figure 3:**
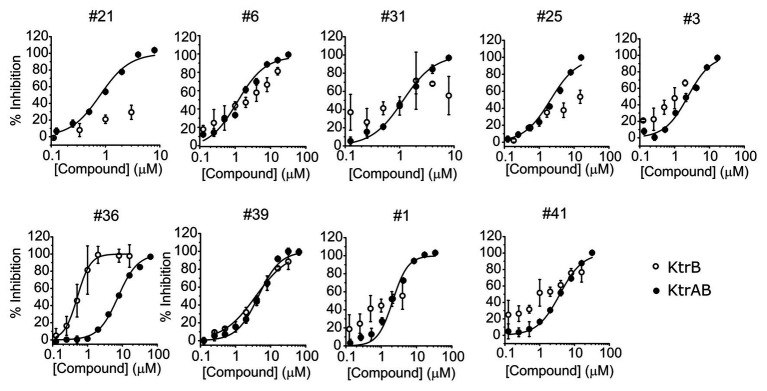
Inhibition of KtrB by representative compounds. Percentage of inhibition of KtrB or KtrAB activity determined using the final fluorescence from flux curves vs. compound concentration, for the indicated compounds. Mean ± SD of triplicates are shown. KtrAB curves were built with the parameters shown in Supplementary Table S1, and KtrB data were fitted with Hill equations with the following parameters: cpd#39, K_I_ = 3.8 μM, n_H_ = 0.92; cpd#36, K_I_ = 0.5 μM, n_H_ = 2.3.

**Figure 4:**
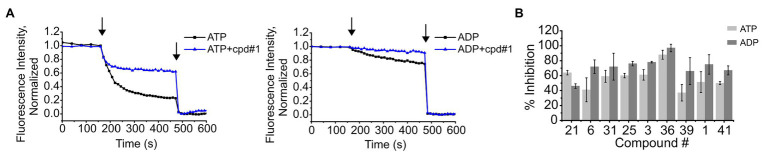
Inhibition of K^+^ flux in the ATP (open) and ADP (closed) forms of KtrAB_R417K_ by representative compounds. **(A)** – Representative curves of K^+^ flux in the presence or absence of 4 μM of cpd #1, in proteoliposomes prepared in the presence of ATP or ADP, as indicated. Arrows indicate addition of CCCP (first) and Val (second). **(B)** – Percentage of inhibition of activity by each compound determined using final fluorescence relative to activity determined in the absence of compound. Compound concentrations used: 4 μM of #1, 6 μM of #36, and 8 μM of the remaining. Mean ± SD of triplicates are shown.

**Figure 5:**
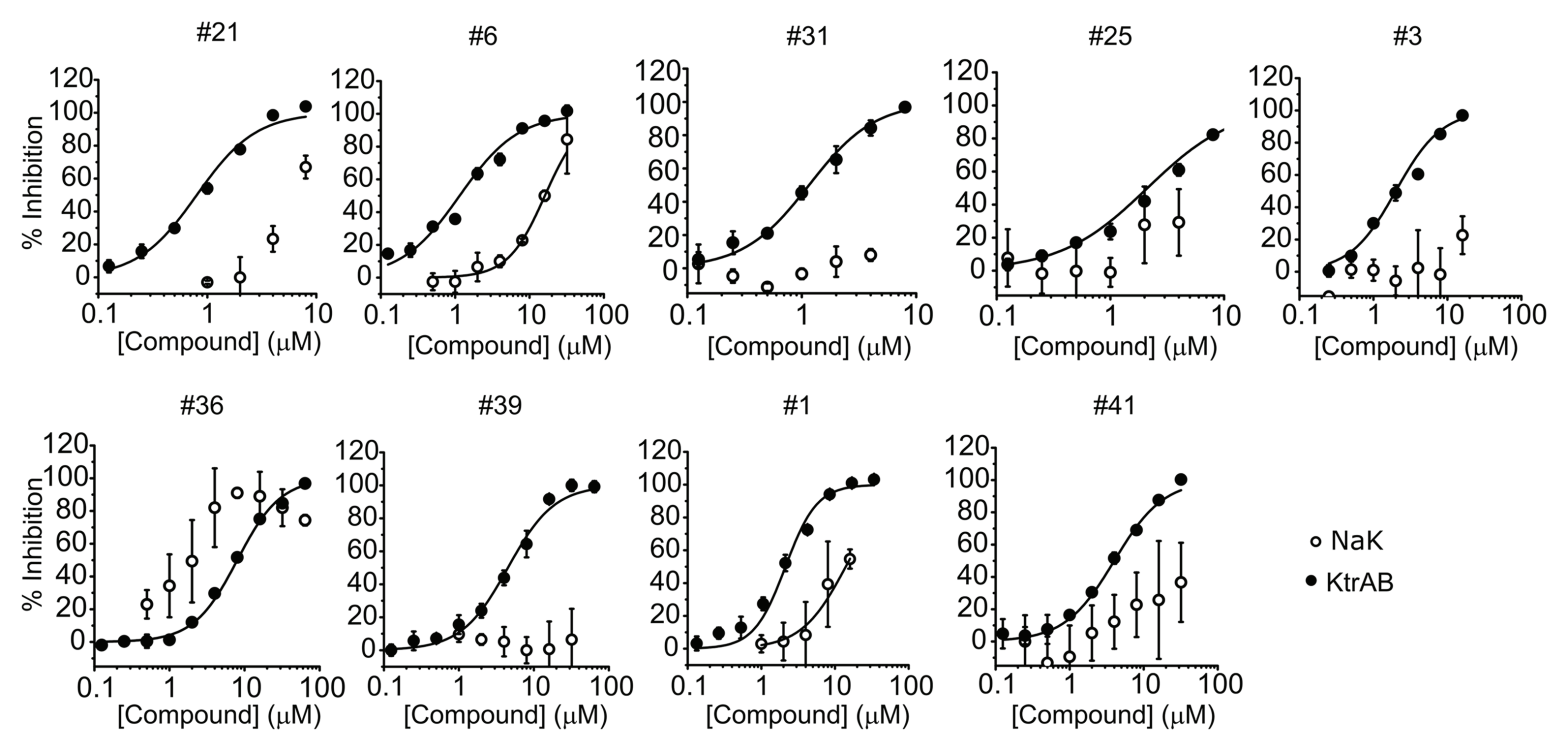
Comparison of inhibition of KtrAB and NaK by representative compounds. Percentage of inhibition of NaK or KtrAB activity was determined using the final fluorescence of flux curves in function of compound concentration, for the indicated compounds. Mean ± SD of triplicates are shown. KtrAB curves were built with the parameters shown in Supplementary Table S1, and NaK data were fit with a Hill equation with the following parameters: #1, K_I_ = 14 μM, n_H_ = 1.4; #6, K_I_ = 16 μM, n_H_ = 1.7.

**Figure 6:**
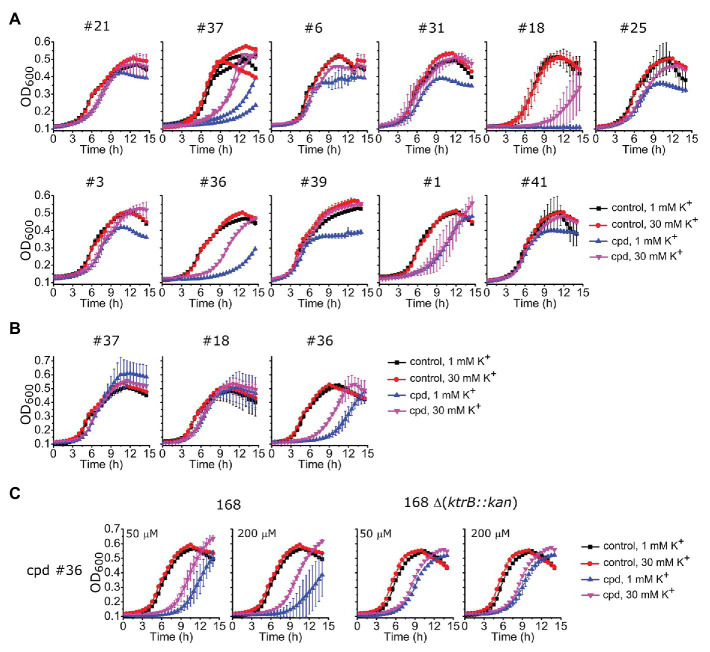
Impact of selected compounds on *Bacillus subtilis* viability. **(A)** – Growth curves of *B. subtilis* JH642 strain at 1 or 30 mM K^+^ for the nine compounds characterized *in vitro* (Table 1) plus other compounds that showed a differential effect, in the absence (control) or presence of the compound (cpd) at the following concentrations: 500 μM of compounds #37 and #18, 300 μM of #36, 300 μM of #6, 100 μM of #31, #25,#3, #39, and #41, 50 μM of #1, and 10 μM of #21. Examples shown correspond to compound concentrations where the clearest effect was observed without total cell arrest, which invariably occurred at higher compound concentrations (Supplementary Figure S5). Supplementary Figure S6 shows growth curves for the remaining 23 compounds tested. **(B)** – Growth curves of *B. subtilis* 168 strain for compounds #37 and #18 under the same conditions as in A whereas compound #36 was 500 μM. **(C)** – Growth curves of *B. subtilis* 168 and 168 *Δ(ktrB::kan)* strains in the presence of compound #36 at the indicated concentrations. In all panels, mean ± SD of triplicates or quadruplicates are shown, except for compound #37 in **(A)**, which shows the results of two independent experiments.

The compound with the lowest K_I_ for KtrAB within each family (Supplementary Table S1; Supplementary Figure S5) was chosen and subjected to the assays in Figures 3–6.

In order to evaluate whether the compounds affect KtrAB K^+^ flux through binding to the cytoplasmic KtrA protein, we measured the thermal stability of purified KtrA protein in the presence or absence of 10 μM of each compound. None of them significantly changed the melting temperature of KtrA (T_m_ = 36.0 ±0.5°C) suggesting that they do not bind to KtrA (data not shown). Due to incompatibility of the thermal shift assay with detergents we could not use this format to evaluate compound effect on the KtrAB complex.

To investigate if the compounds affect KtrAB K^+^ flux directly through the membrane protein KtrB, we performed the K^+^ flux assay in liposomes containing the membrane subunit KtrB only. In the absence of the regulatory octameric KtrA ring, K^+^ flux is reduced 10-fold but a small K^+^ efflux can still be observed (Teixeira-Duarte et al., 2019). We, therefore, tested the functional effect of one compound from each structural group (Table 1) on KtrB alone (Figure 3). We observed inhibition of K^+^ efflux with the majority of compounds tested, but quantification of the effect was difficult due to the low activity of KtrB (Figure 3). Interestingly, compound #36, a urea derivative, showed a significantly higher potency of inhibition on KtrB K^+^ efflux (K_I_ = 0.5 μM) compared to that seen on the KtrAB complex (K_I_ = 8 μM).

We have previously shown that K^+^ flux through KtrAB is conformation or state-dependent (Vieira-Pires et al., 2013; Szollosi et al., 2016; Teixeira-Duarte et al., 2019). Up to this point, we have been assaying compound inhibition of K^+^ flux through the KtrAB complex in the active or open state, in the presence of ATP. In order to understand if compound binding and inhibitory effect is limited to one particular conformation, we tested the capacity of the compounds also to inhibit the inactive conformation, in the presence of ADP. This was achieved by measuring K^+^ flux using a previously characterized mutant of KtrAB, KtrAB_R417K_, in the presence of ADP. The KtrAB_R417K_-ADP mutant is thought to still acquire the inactive conformation of wild type KtrAB-ADP while exhibiting a higher K^+^ flux, making it more amenable to assay (Szollosi et al., 2016). As shown in Figures 4A,B, all tested compounds showed inhibition with both the active and inactive forms of KtrAB_R417K_ leading us to conclude that binding of the inhibitors is not state-dependent.

To evaluate the specificity of compounds for the KtrAB channel, we chose to look at the inhibitory effect of the same subset of nine representative compounds on the NaK cation channel from *B. cereus*. This protein does not belong to the SKT superfamily; however, it does have a similar membrane structural architecture. We used the NaK_*Δ*N19/F92A_ mutant form of the channel in our flux liposome assay, as it has been reported to show higher activity than the wild type form (Alam and Jiang, 2009). As shown in Figure 5, the majority of compounds had lower efficacy against the NaK_ΔN19/F92A_ channel (with at least 10 × higher K_I_), and compound #39 showed no inhibition within the tested concentration range. This data suggests that these compounds are specific in their action on the KtrAB channel. Notably, compound #36, which affected KtrAB activity through the membrane protein, KtrB, also showed a similar inhibitory effect on the NaK channel.

### Impact on Cell Viability

To investigate the action of these identified compounds on cell viability through disruption of K^+^ homeostasis, we utilized the fact that KtrAB is required for *B. subtilis* growth at low K^+^ concentrations (Holtmann et al., 2003). We expected that compounds that inhibited *B. subtilis* KtrAB activity in liposomes would also inhibit *B. subtilis* cell growth in low K^+^ (1 mM) but not in high K^+^ (30 mM). Initially, we tested the *B. subtilis* strain JH642 as it is devoid of the KimA K^+^ importer (Gundlach et al., 2017) and as such is dependent on KtrAB for growth in low K^+^ conditions. This strain was grown in low and high K^+^ media in the presence of different concentrations of the compounds that showed an *in vitro* inhibitory dose-response with K_I_ ≤ 10 μM (34 compounds). Beyond a certain concentration threshold, we observed complete inhibition of growth with all compounds that may also result from off target effects. We focused on intermediate concentrations where we could look for a K^+^ specific growth phenotype. Three compounds markedly showed an inhibition of growth at 1 mM K^+^ compared with 30 mM K^+^: #37, belonging to the benzimidazol group, #18 from the piperidine group, and #36 from the urea group (Figure 6A; Supplementary Figures S5, S6). The inhibitory effect is modest, with high concentrations of compound (100–500 μM) required.

To further test the efficacy of compounds #37, 18 and 36 we also evaluated their impact on the parental *B. subtilis* strain 168, which, in addition to the KtrAB channel contains the KimA K^+^ importer. Compound #36 maintained the same phenotype, showing an extended delay in cell growth in 1 mM K^+^ that was partially compensated in 30 mM K^+^ (Figure 6B), while the effect disappeared for compounds #37 and #18. Moreover, the amplitude of the K^+^ differential effect observed for compound #36 was concentration dependent, increasing with compound concentration (Figures 6B, left panel of C). The differential effect was abolished in a mutant strain that lacks KtrAB, strain 168 ∆ (*ktrB::kan*), confirming that compound #36 is targeting KtrAB (Figure 6C, right panel).

## Discussion

We have successfully executed a fluorescence-based liposome K^+^ flux assay to identify novel molecules that inhibit the KtrAB channel from *B. subtilis*. First, this liposome assay is very robust; it is easily adapted to a plate format for screening campaigns, and it allows screening with reduced automation coupled to a relatively simple low time-resolution plate reader. The liposome flux assay can also be used for the follow-up stages, estimation of compound potency and confirmation of liposome integrity. Second, we have identified a number of chemical groups that inhibit KtrAB activity *in vitro* with moderate potency, in the μM range.

Among the compounds identified, #36 (a urea derivative) was unique as it showed K^+^-dependent cell growth inhibition in the mutant strain JH642 (where KtrAB is essential for growth in low K^+^ concentrations) and in strain 168, which contains other K^+^ importers besides KtrAB. Functional characterization of compound #36 showed that KtrAB inhibition most likely results from binding to the membrane protein (KtrB). It also revealed *in vitro* inhibition of the NaK channel (a cation channel from *B. cereus*) with similar potency. Although KtrAB and NaK have very different amino acid sequences, they are both non-selective monovalent cation channels with ion pores that adopt the TM-Ploop-TM architecture. We speculate that compound #36 may bind to and recognize a structural feature present in both channels. This is not uncommon, as a number of studies have shown that molecules can bind to a membrane-buried crevice positioned between subunits or repeats of the ion pore of TM-Ploop-TM channels with very different sequences (Finol-Urdaneta et al., 2019; Zhu et al., 2020). Importantly, knockout of KtrB from strain 168 abolished the K^+^-dependent inhibition by compound #36, demonstrating specific targeting of KtrAB in the cell.

The K^+^-dependent phenotype associated with compound #36 may appear subtle. However, this is a striking result as it reveals that inhibition of a specific component of the bacterial K^+^ homeostasis machinery has an impact on bacterial growth, even when there is apparent functional redundancy due to the presence of other K^+^ transporters. Importantly, the K^+^-dependent phenotype observed with inhibited cells strongly resembles that of mutant strains with deficient K^+^ transport (Holtmann et al., 2003), which display perturbed growth under environmental challenges (for example, low K^+^ or osmotic stress). Bacteria meet this type of challenges at different stages of the process of infection (Fang et al., 2016; Janakiraman and Lesser, 2017), supporting our purpose of establishing a screening strategy for compounds that target components of the K^+^ homeostasis machinery. In addition, the high inhibitory concentrations required (100–500 μM) indicate that compound #36 displays modest potency. This feature may be resolved through chemical expansion, using a targeted medicinal chemistry approach. In fact, urea derivatives are present in a number of pharmacological drugs, are available commercially, and have shown antimicrobial activity (Patil et al., 2019a,b).

In conclusion, this proof-of-concept study opens the door for expanded screening campaigns, covering a larger chemical space, targeting conserved components of the K^+^ homeostasis machinery. With few exceptions, the current antibiotics have the same few cellular targets (cell wall, protein biosynthesis, and DNA biosynthesis). Being excluded from animal cells and essential to *S. aureus* and other bacteria with a marked role in virulence, antibacterial resistance and adaptation to environmental changes, SKT members are promising alternative targets for antibiotic development.

## Data Availability Statement

The original contributions presented in the study are included in the article/Supplementary Material, further inquiries can be directed to the corresponding author.

## Author Contributions

JM-C, CH, and AF designed the experiments. AF, AP, and CT-D performed the experiments. AF and CH analyzed the data. JM-C, CH, AF, AP, and CT-D wrote the manuscript. All authors contributed to the article and approved the submitted version.

### Conflict of Interest

The authors declare that the research was conducted in the absence of any commercial or financial relationships that could be construed as a potential conflict of interest.
