# Supplementary material for: Fluorometric Liposome Screen for Inhibitors of a Physiologically Important Bacterial Ion Channel
Source: Front Microbiol. 2021 Mar 1;12:603700. doi: 10.3389/fmicb.2021.603700 (PMC7956971; doi:10.3389/fmicb.2021.603700)
Supplement: Supplementary file 1 [file Data_Sheet_1.pdf]

Supplementary Material

Figure S1

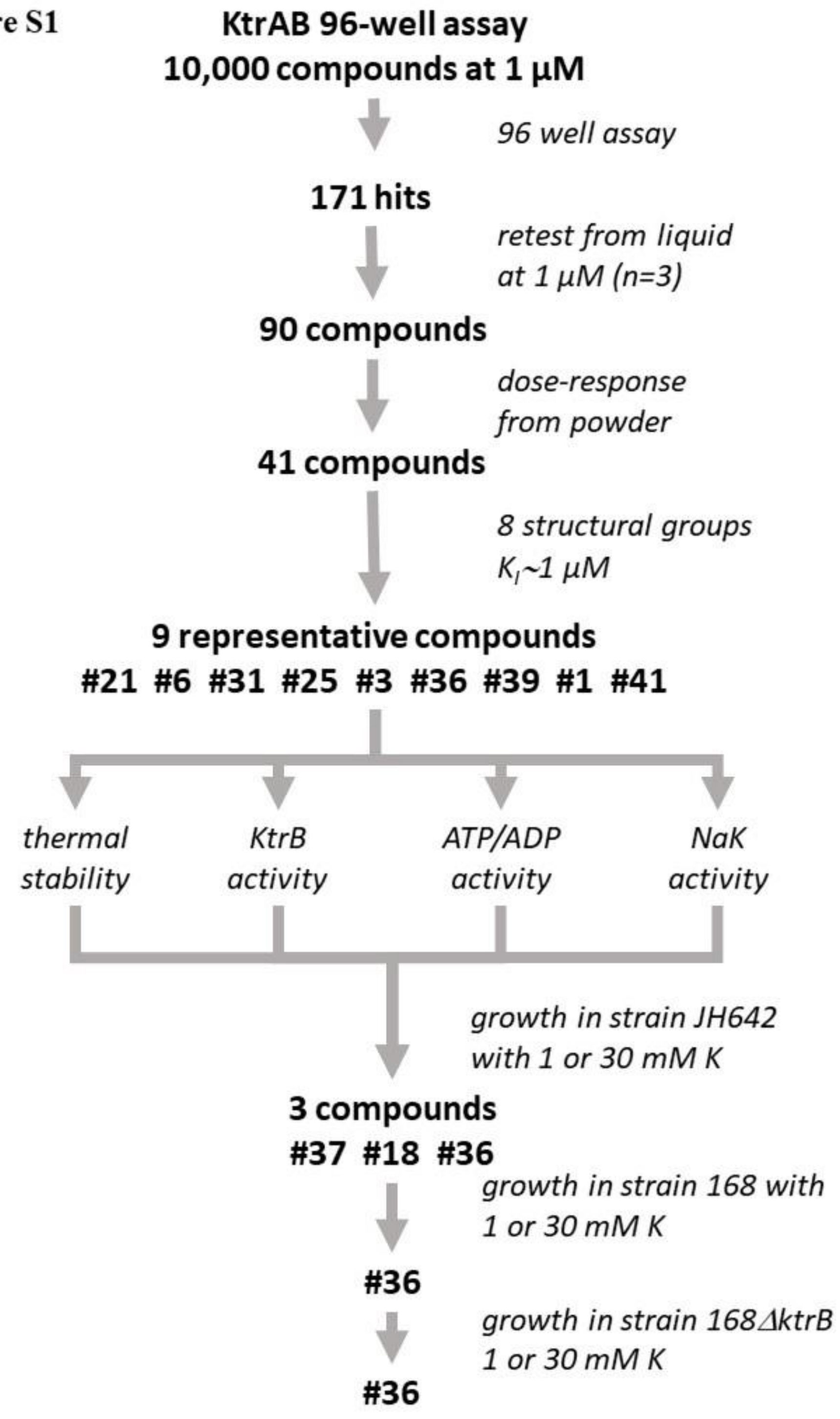

**Figure S2**

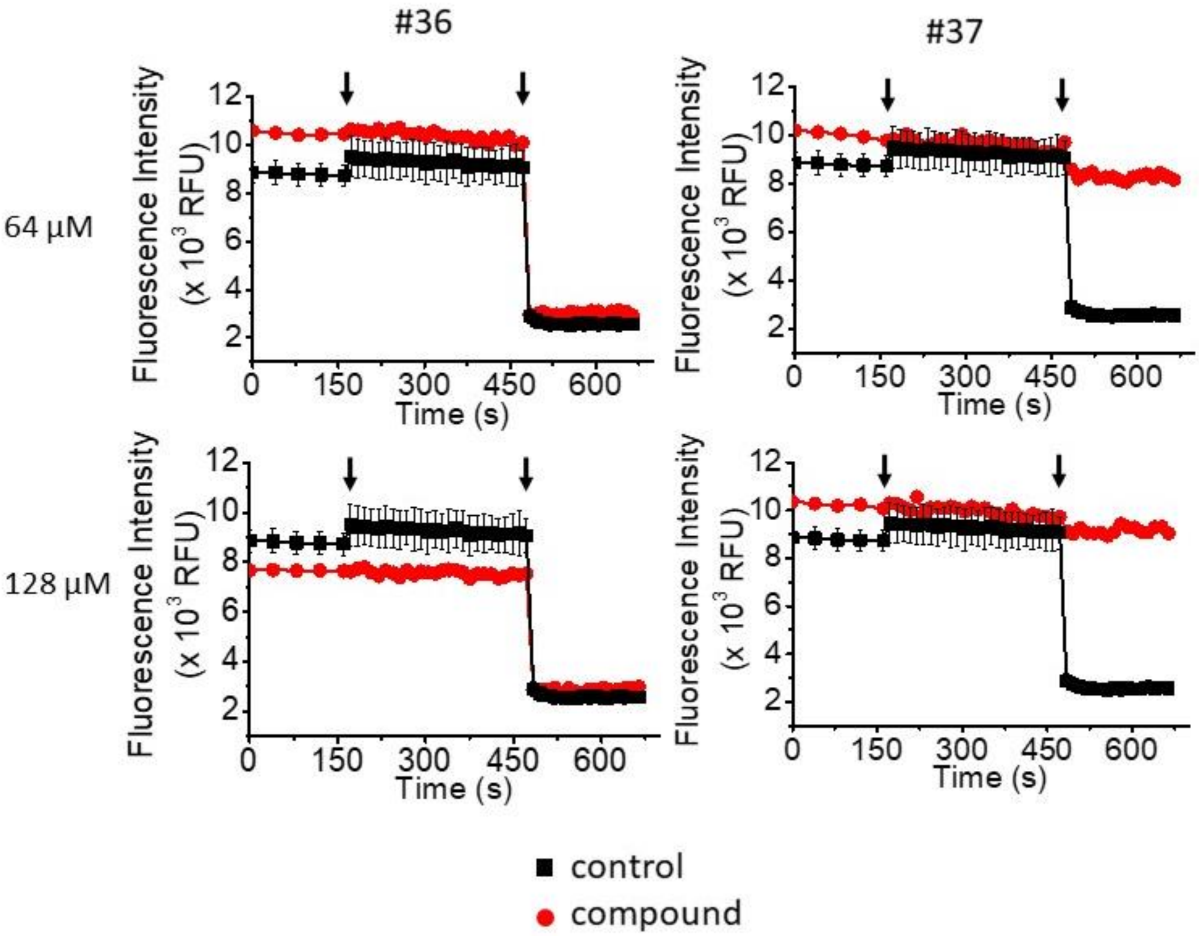


**Figure S2** - Curves of $K^+$ flux through empty liposomes in the presence or absence of the indicated concentrations of compounds #36 or #37. Arrows indicate addition of CCCP (first) and valinomycin (second).

**Figure S3 (page 1/3)**

## Benzimidazols

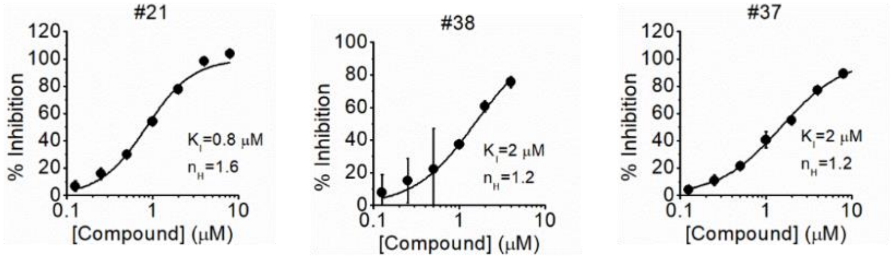


## Piperazines

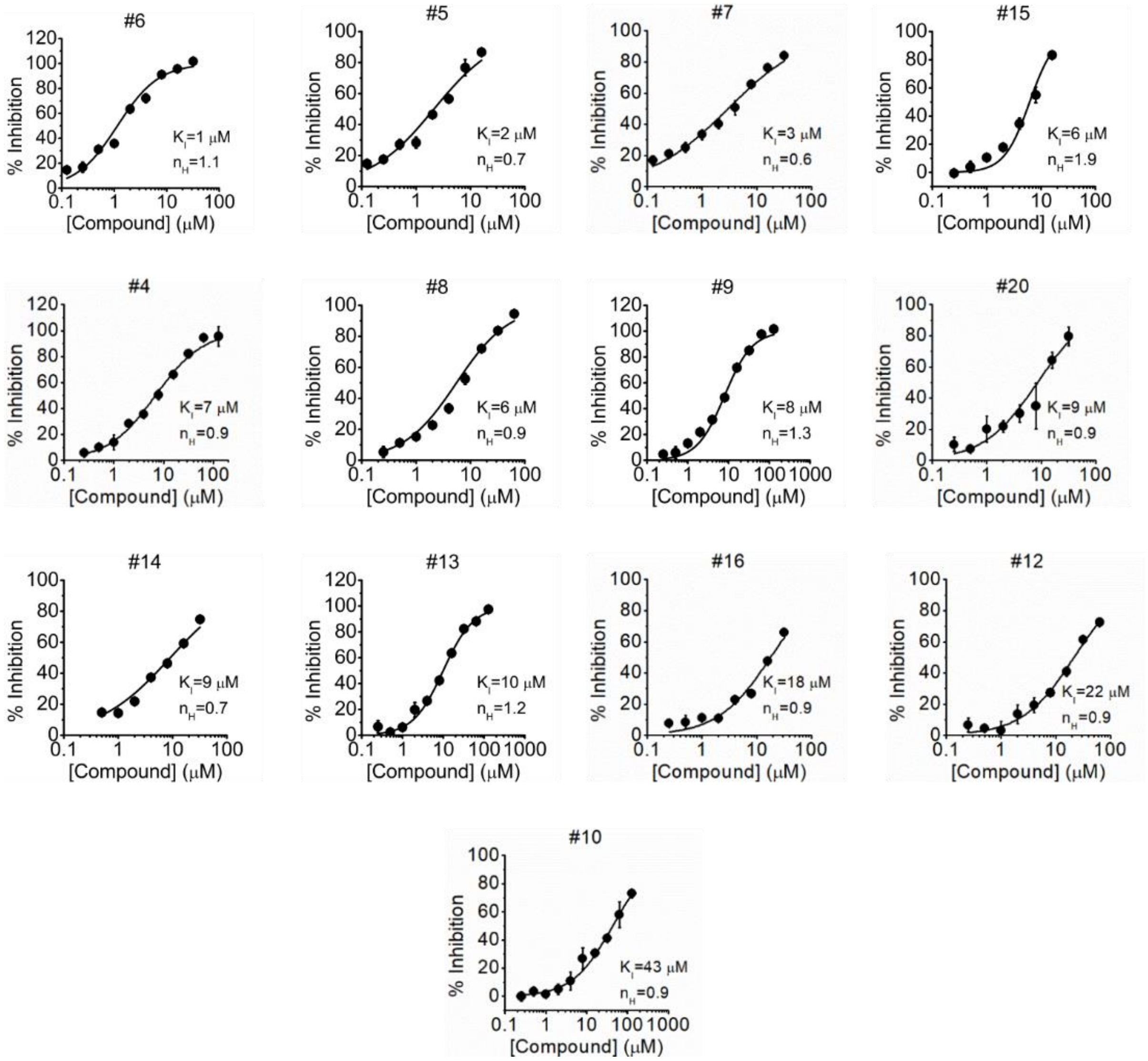

**Figure S3 (page 2/3)**

## Piperidines

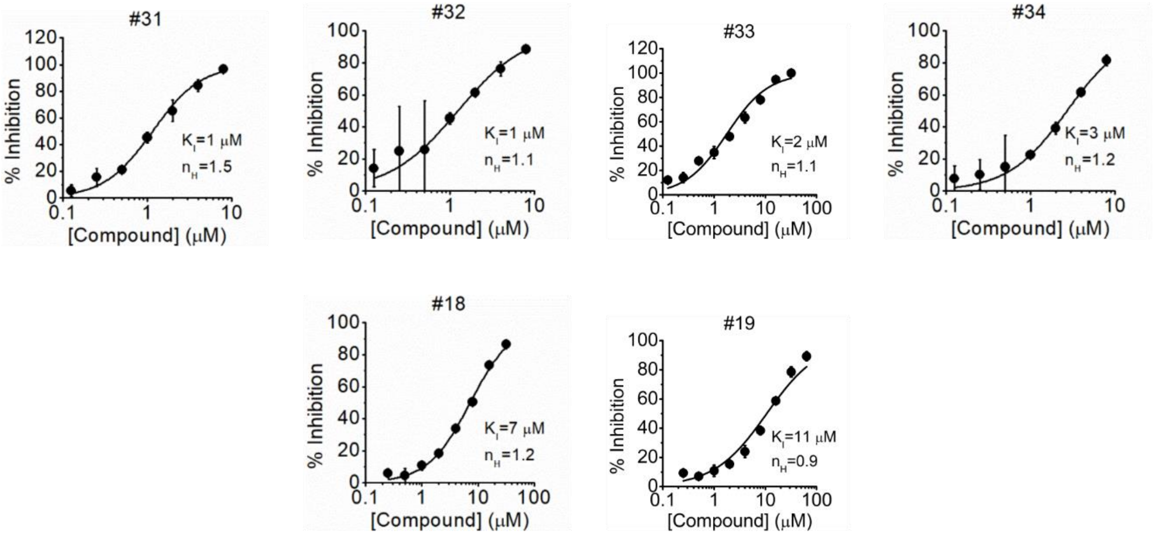


## Phenoxybutyl piperidines and related

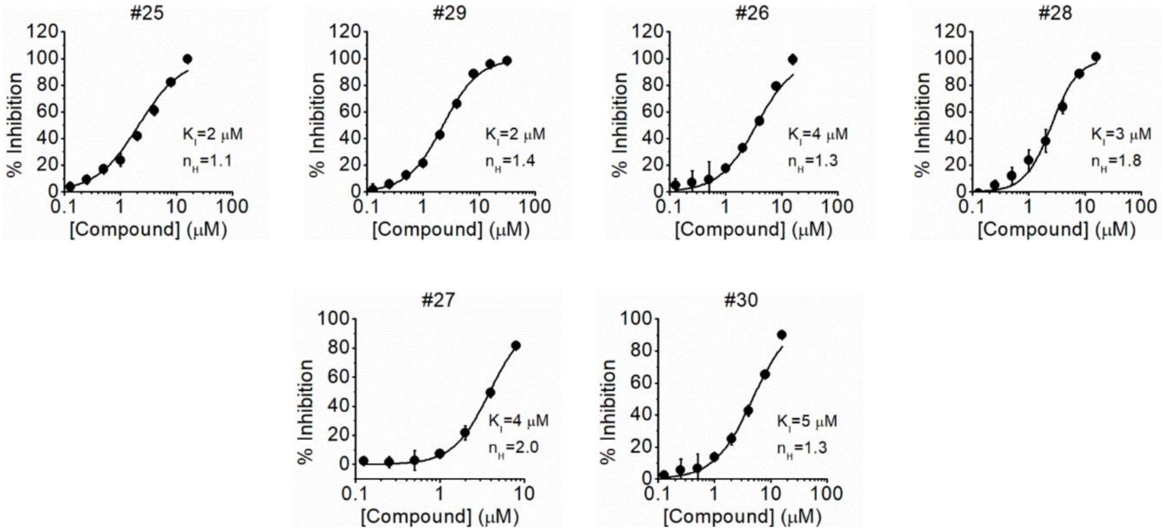


## Azepanes

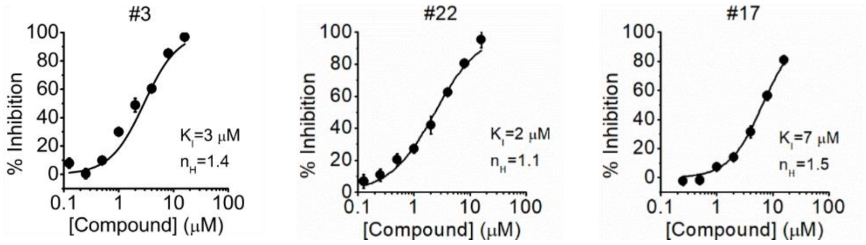

**Figure S3 (page 3/3)**

## Ureas

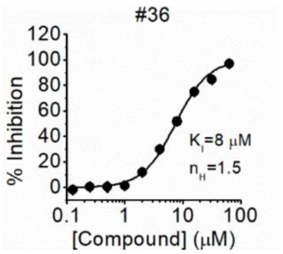


## Phenylethylamines and related

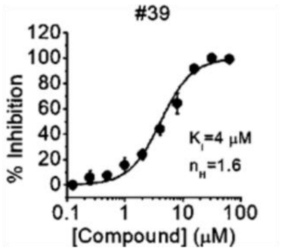


## Others

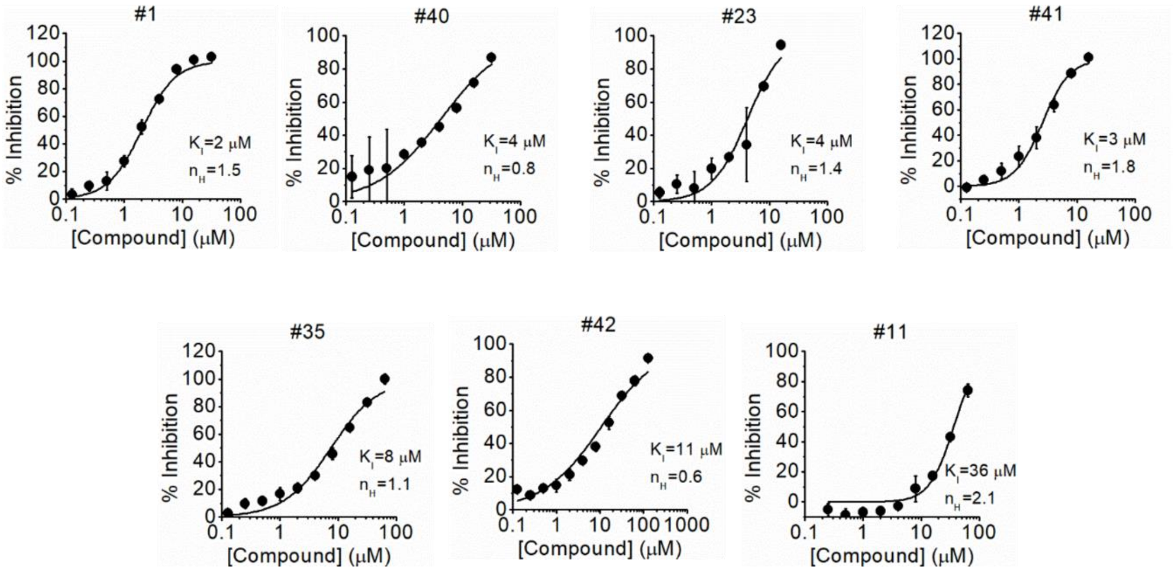


**Figure S3 – Dose-response of all compounds from the different families.** Percentage of inhibition of KtrAB activity determined using the final fluorescence from $K^+$ flux assays performed in 96-well plates as function of compound concentration. Mean ± standard deviation of triplicates are shown fitted with Hill equation and indicated $n_H$ and $K_I$ parameters.

**Figure S4**

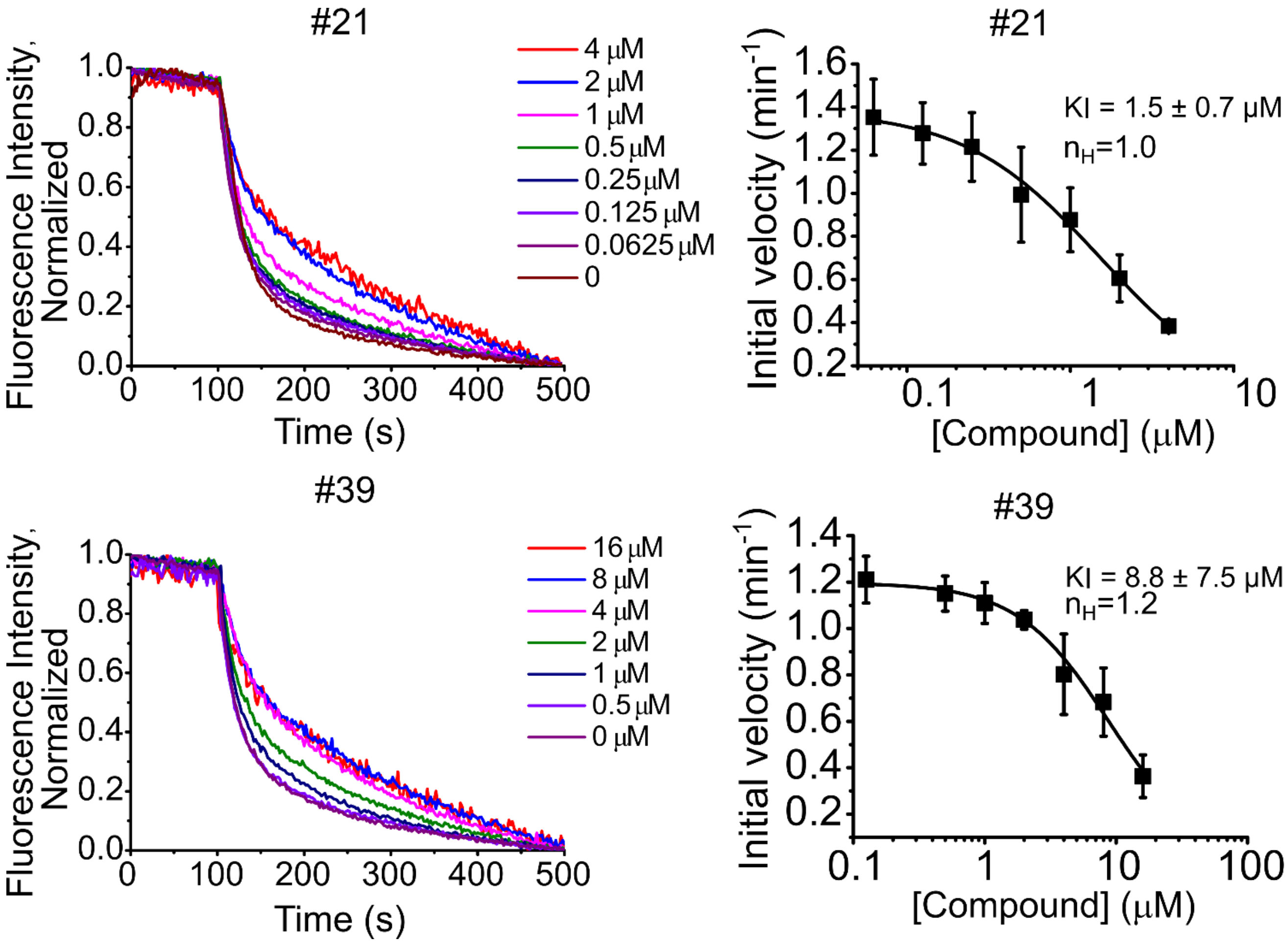


**Figure S4 - Cuvette dose-response curves normalized for final fluorescence. Left** - Representative experiments of $K^+$ flux assays performed in cuvette with the indicated concentrations of compound #21 (top) or #39 (bottom). Curves were normalized using as maximum the initial fluorescence (before CCCP addition) and as minimum fluorescences values immediately before valinomycin addition. **Right** - Initial velocities determined from curves (on the left) as function of compound concentration, for compound #21 (top) or #39 (bottom). Mean ± standard deviation of triplicates are shown fitted with a Hill equation and the indicated $n_H$ and $K_I$ ± standard error parameters.

**Figure S5 (page 1/2)**

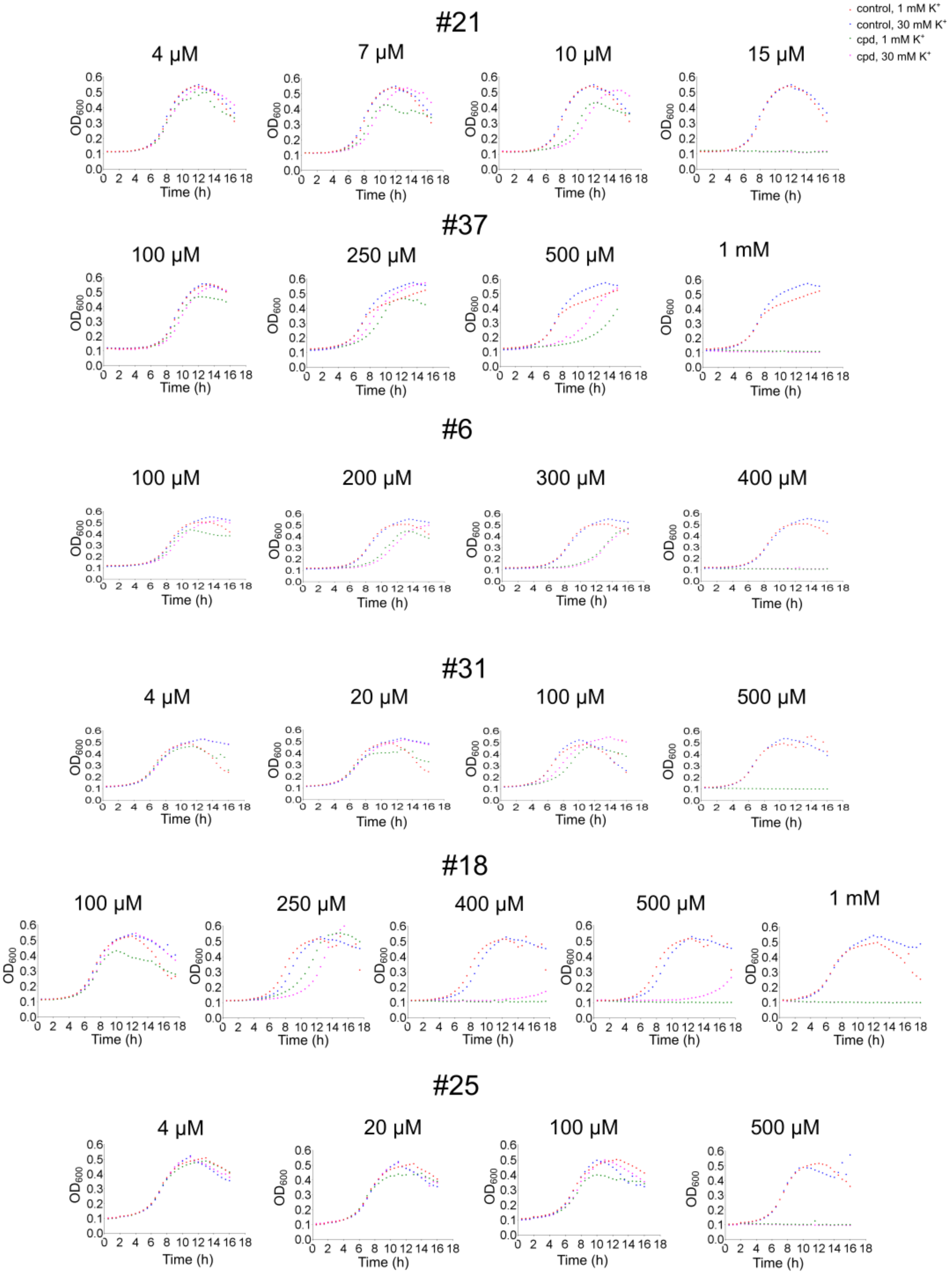

**Figure S5 (page 2/2)**

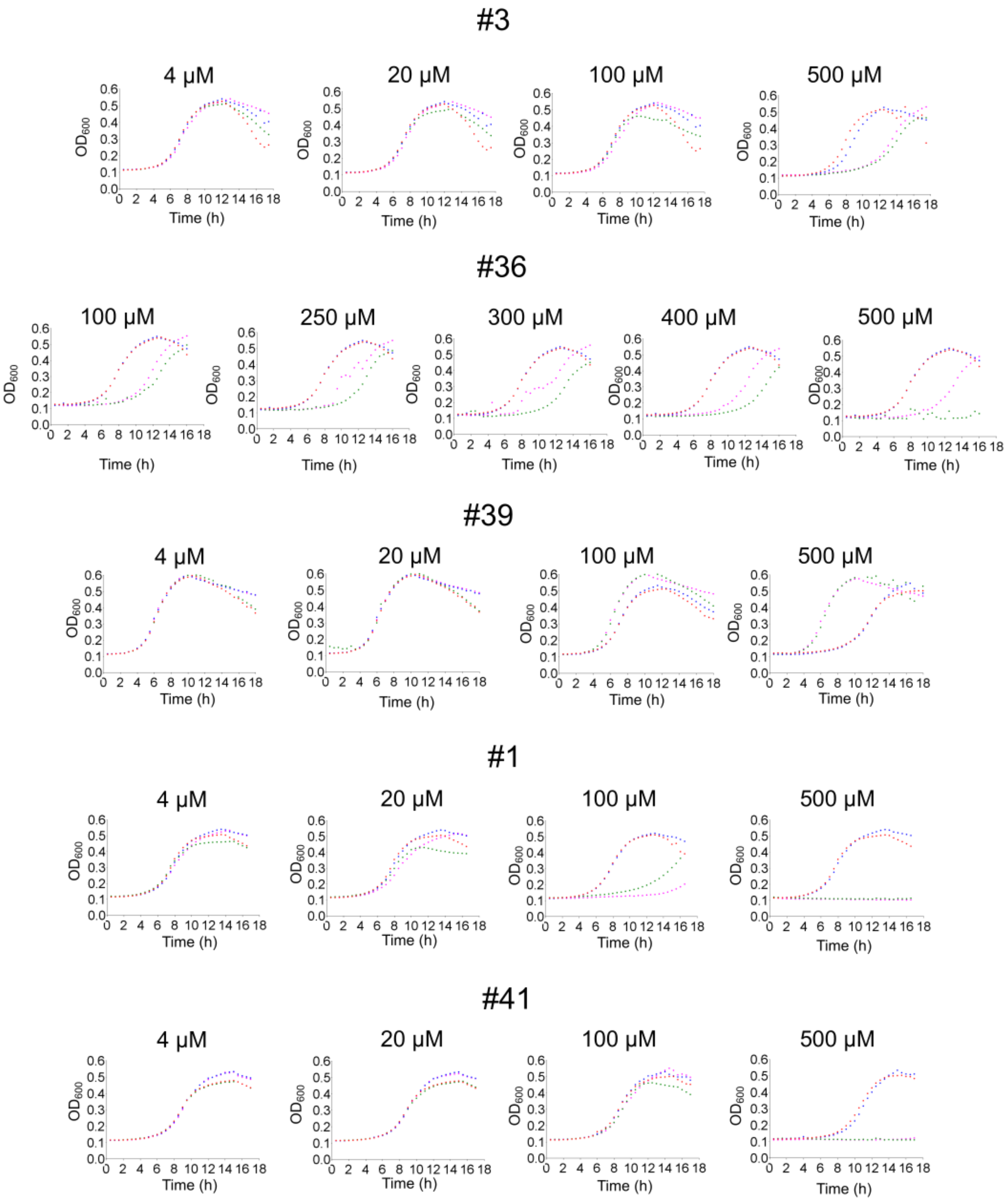


**Figure S5 – Full set of growth curves of *B. subtilis* JH642 strain.** Example of growth curves of JH642 at 1 or 30 mM $K^+$, in the absence (control) or presence of the indicated compounds (cpd) at the indicated concentrations.

**Figure S6 (page 1/2)**

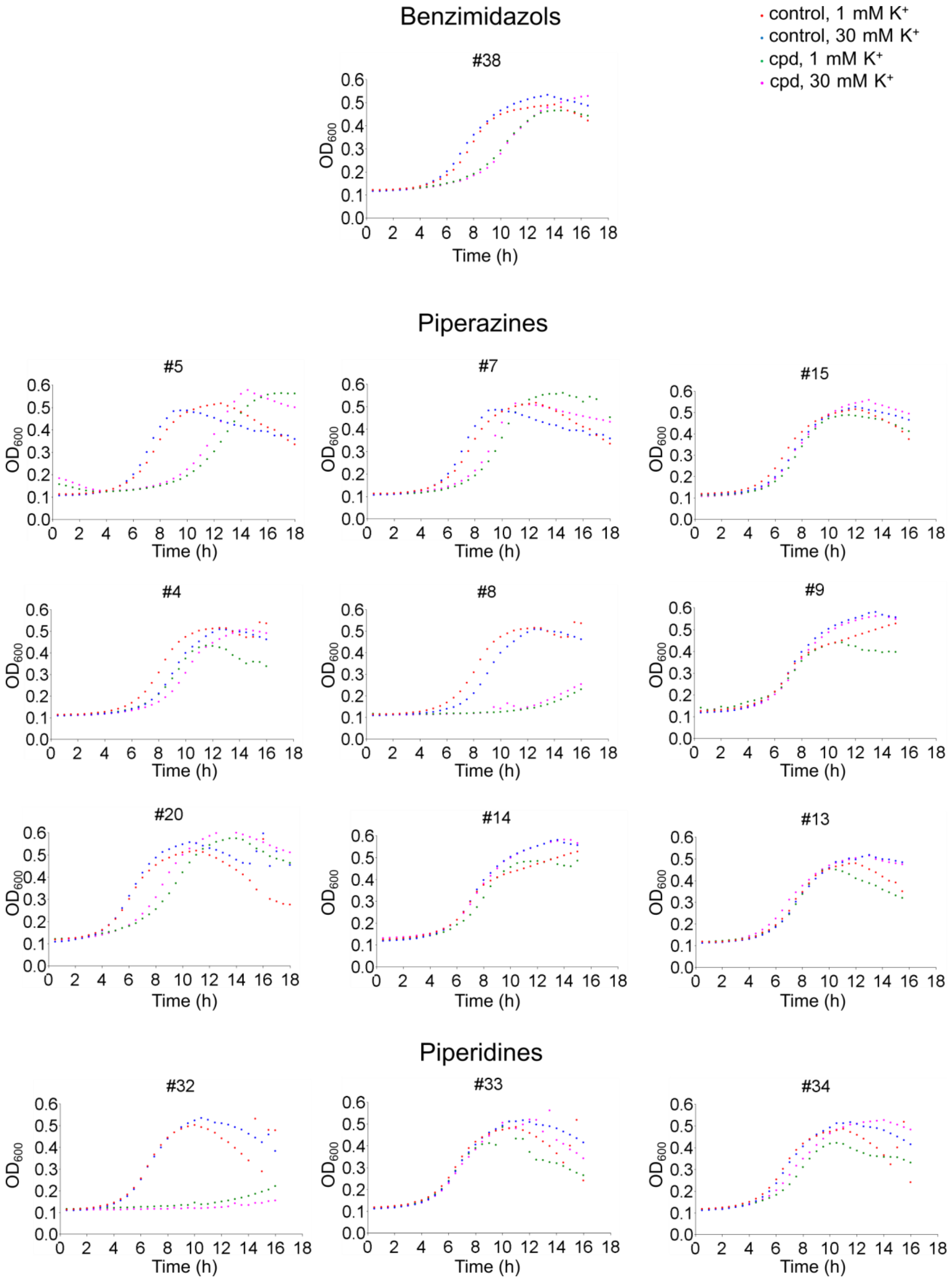

**Figure S6 (page 2/2)**

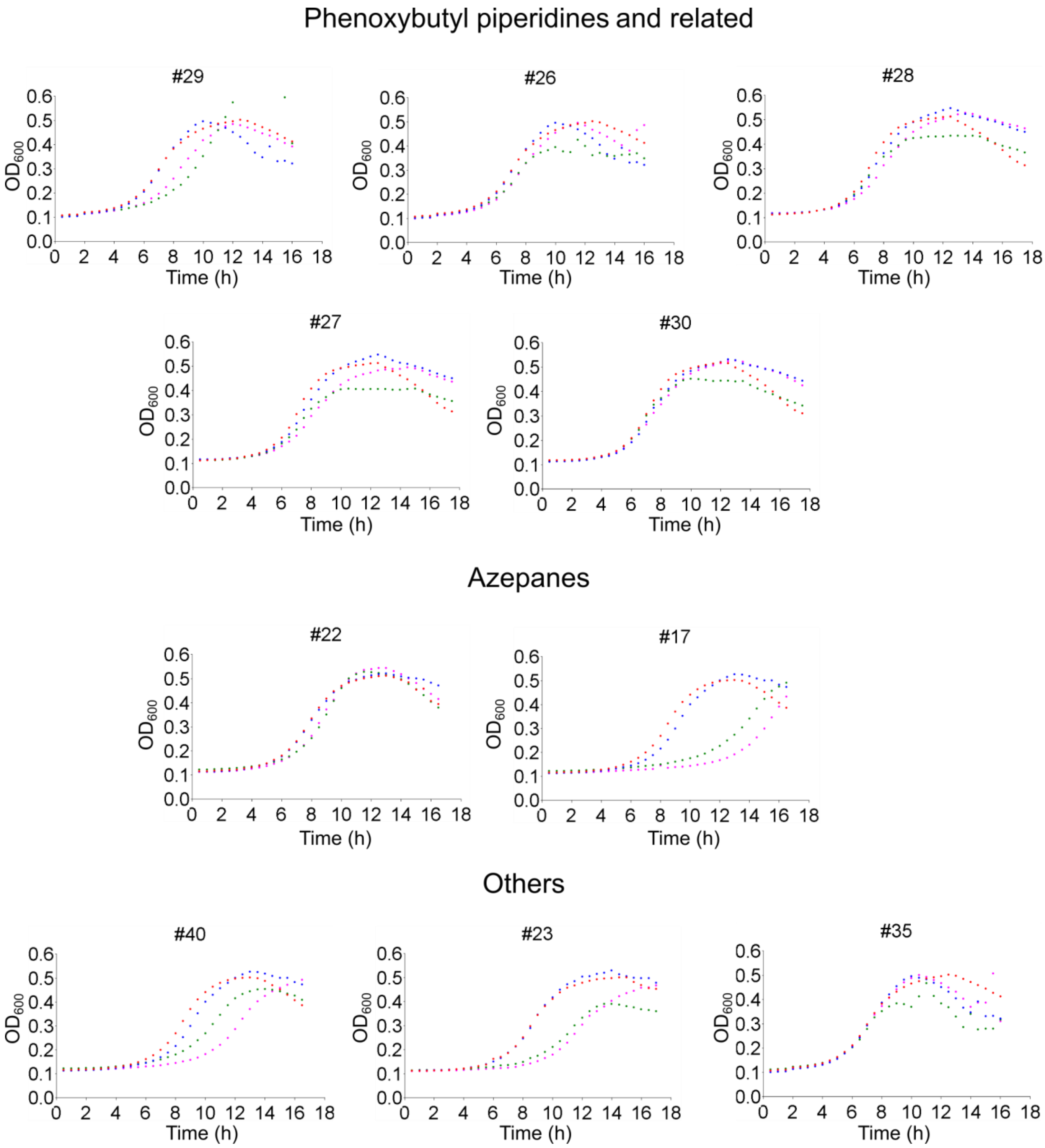


**Figure S6** - Growth curves of *B. subtilis* JH642 strain at 1 or 30 mM $K^+$, in the absence (control) or presence of the indicated compounds (cpd) at the following concentrations: 500 µM of compounds #5, 7, 4, 8, 9, 20, 14, 13, 32, 17, and 40, 300 µM of #38, 100 µM of #15, 33, 34, 35, 29, 26, 22, and 23, and 20 µM of #28, 27, and 30.

**Table S1 (page 1/5)**

| cpd # | Strucutural Group | Name | cpd ID | Structure | $K_I$ (μM) | $n_H$ |
|---|---|---|---|---|---|---|
| 21 | Benzimidazols | 1-(1-adamantyl)-2-{3-[2-(diethylamino)ethyl]-2-imino-2,3-dihydro-1H-benzimidazol-1-yl}ethanone hydrobromide | 5283510 | 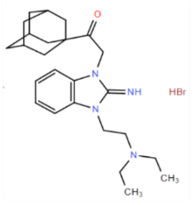 | 0.8 ± 0.04 | 1.6 |
| 38 | | 2-{2-imino-3-[2-(1-piperidinyl)ethyl]-2,3-dihydro-1H-benzimidazol-1-yl}-1-phenylethanol hydrochloride | 5784716 | 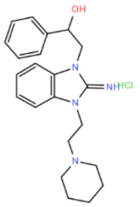 | 2 ± 0.03 | 1.2 |
| 37 | | 2-{2-imino-3-[2-(1-piperidinyl)ethyl]-2,3-dihydro-1H-benzimidazol-1-yl}-1-(2-thienyl)ethanol hydrochloride | 5786308 | 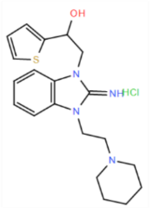 | 2 ± 0.1 | 1.2 |
| 6 | Piperazines | 1-(2,4-dimethoxybenzyl)-4-(2,3-dimethylphenyl)piperazine | 5257793 | 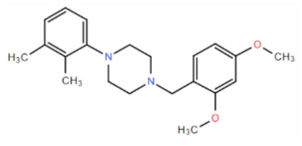 | 1 ± 0.2 | 1.1 |
| 5 | | 1-(2,3-dimethylphenyl)-4-(4-methoxy-3-methylbenzyl)piperazine | 5257553 | 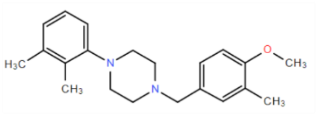 | 2 ± 0.2 | 0.7 |
| 7 | | 1-(2-ethoxybenzyl)-4-(2-methylphenyl)piperazine | 5258265 | 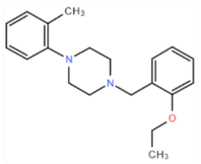 | 3 ± 0.2 | 0.6 |
| 15 | | 1-(4-biphenylylmethyl)-4-ethylpiperazine | 5262889 | 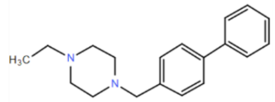 | 6 ± 1 | 1.9 |
| 4 | | 1-(2-methylphenyl)-4-[4-(methylthio)benzyl]piperazine | 5257279 | 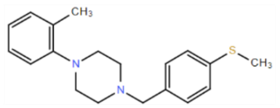 | 7 ± 0.5 | 0.9 |
| 8 | | 1-(4-methoxybenzyl)-4-(2-methylphenyl)piperazine oxalate | 5258361 | 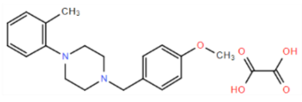 | 6 ± 0.5 | 0.9 |

**Table S1 (page 2/5)**

| cpd # | Structural Group | Name | cpd ID | Structure | $K_I$ (μM) | $n_H$ |
|---|---|---|---|---|---|---|
| 9 | | 1-(4-chlorophenyl)-4-(2-ethoxybenzyl)piperazine | 5258935 | 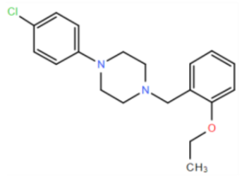 | 8 ± 0.4 | 1.3 |
| 20 | | 1-(2-methylbenzyl)-4-(3-phenyl-2-propen-1-yl)piperazine | 5270678 | 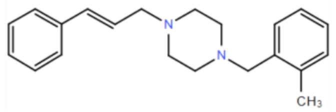 | 9 ± 1 | 0.9 |
| 14 | | 1-(4-ethylbenzyl)-4-(2-fluorophenyl)piperazine | 5262873 | 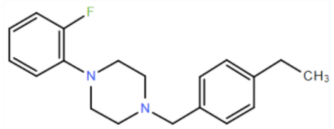 | 9 ± 2 | 0.7 |
| 13 | Piperazines | 1-(1,3-benzodioxol-5-ylmethyl)-4-(4-ethylbenzyl)piperazine | 5262635 | 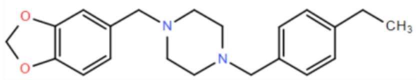 | 10 ± 0.5 | 1.2 |
| 16 | | 1-(2-ethoxybenzyl)-4-[3-(trifluoromethyl)phenyl]piperazine | 5265556 | 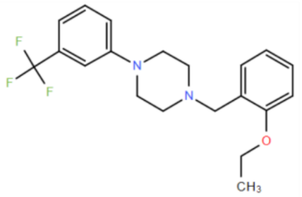 | 18 ± 2 | 0.9 |
| 12 | | (4-{[4-(2,3-dimethylphenyl)-1-piperazinyl]methyl}phenyl)dimethylamine | 5260949 | 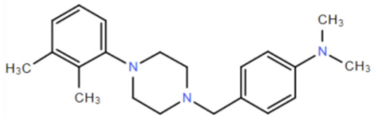 | 22 ± 1 | 0.9 |
| 10 | | 1-(2,3-dimethylphenyl)-4-(2-methoxybenzyl)piperazine | 5259050 | 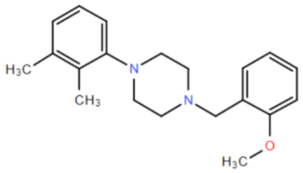 | 43 ± 2 | 0.9 |
| 31 | Piperidines | N-benzyl-N-(2-phenylethyl)-1-propyl-4-piperidinamine | 5454352 | 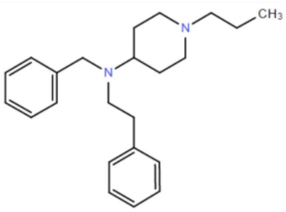 | 1 ± 0.06 | 1.5 |

**Table S1 (page 3/5)**

| cpd # | Strucutural Group | Name | cpd ID | Structure | $K_I$ (μM) | $n_H$ |
|---|---|---|---|---|---|---|
| 32 | Piperidines | 1-(2-isopropyl-5-methylphenoxy)-3-(3-methyl-1-piperidinyl)-2-propanol hydrochloride | 5769795 | 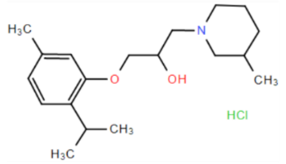 | 1 ± 0.05 | 1.1 |
| 33 | | 1-(4-isopropyl-3-methylphenoxy)-3-(3-methyl-1-piperidinyl)-2-propanol hydrochloride | 5747926 | 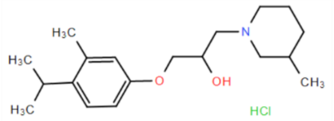 | 2 ± 0.3 | 1.1 |
| 34 | | 1-(1-piperidinyl)-3-(1,2,3,4-tetrahydro-9H-carbazol-9-yl)-2-propanol | 5679709 | 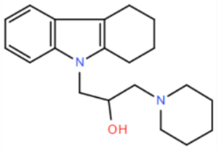 | 3 ± 0.05 | 1.2 |
| 18 | | 4-benzyl-1-(3,4-dimethoxybenzyl)piperidine | 5269603 | 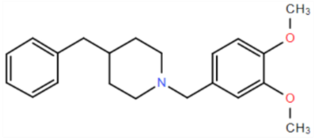 | 7 ± 0.3 | 1.5 |
| 19 | | ethyl 1-(4-biphenylylmethyl)-3-piperidinecarboxylate | 5269778 | 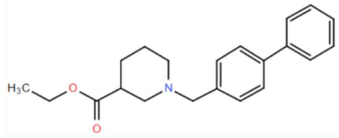 | 11 ± 1 | 0.9 |
| 25 | Phenoxybutyl piperidines and related | 1-[4-(2,3,5-trimethylphenoxy)butyl]piperidine | 5364692 | 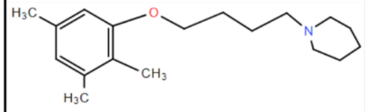 | 2 ± 0.03 | 1.1 |
| 29 | | 1-[4-(1-naphthyloxy)butyl]piperidine | 5358914 | 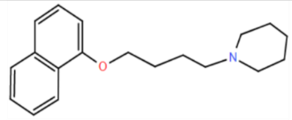 | 2 ± 0.1 | 1.4 |
| 26 | | 1-[4-(4-chlorophenoxy)butyl]piperidine | 5366651 | 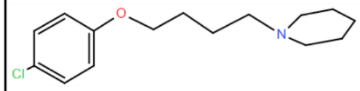 | 4 ± 0.1 | 1.3 |
| 28 | | 1-[4-(2-tert-butylphenoxy)butyl]pyrrolidine | 5360459 | 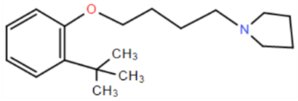 | 3 ± 0.1 | 1.8 |
| 27 | | 1-{2-[(4-chloro-1-naphthyl)oxy]ethyl}pyrrolidine | 5468663 | 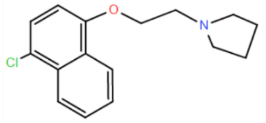 | 4 ± 0.2 | 2.0 |

**Table S1 (page 4/5)**

| cpd # | Structural Group | Name | cpd ID | Structure | $K_I$ (μM) | $n_H$ |
|---|---|---|---|---|---|---|
| 30 | Phenoxybutyl piperidines and related | 1-[4-(2-chloro-4-methylphenoxy)butyl]pyrrolidine | 5536673 | 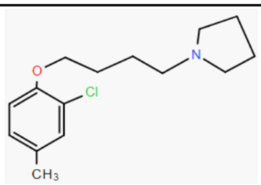 | 5 ± 0.2 | 1.3 |
| 3 | Azepanes | 1-(1-azepanyl)-3-(diphenylamino)-2-propanol hydrochloride | 5236886 | 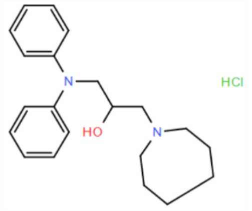 | 2 ± 0.2 | 1.4 |
| 22 | | 1-[4-(benzyloxy)benzyl]azepane | 5426168 | 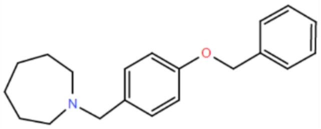 | 2 ± 0.1 | 1.1 |
| 17 | | 1-(3,4-dichlorobenzyl)-4-methyl-1,4-diazepane | 5269275 | 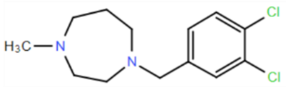 | 7 ± 0.6 | 1.6 |
| 36 | Ureas | N-(4-chlorophenyl)-N'-[3-(trifluoromethyl)phenyl]urea | 5359467 | 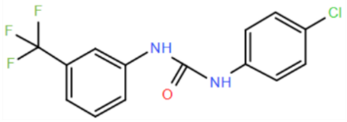 | 8 ± 0.2 | 1.5 |
| 39 | Phenylethylamines and related | (5-bromo-2-methoxybenzyl)[2-(3-fluorophenyl)ethyl]amine | 5538912 | 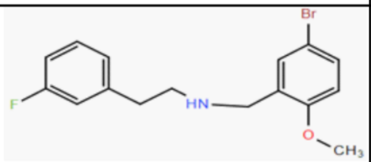 | 4 ± 0.3 | 1.6 |
| 24 | Hydrazones | 2-pyridinecarbaldehyde 1,3-benzothiazol-2-ylhydrazone | 5633522 | 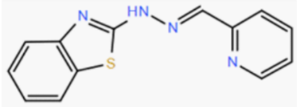 | | |
| 1 | Others | N-[2-hydroxy-3-(mesityloxy)propyl]-2,4,4-trimethyl-2-pentanaminium chloride | 5144511 | 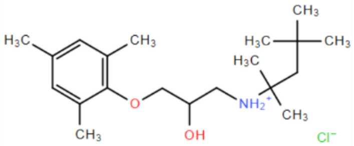 | 2 ± 0.3 | 1.5 |
| 40 | | N-benzyl-N-ethyl-3-(2-methoxyphenyl)-2-propen-1-amine | 5431552 | 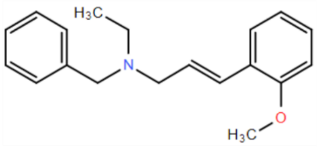 | 4 ± 0.4 | 0.8 |

**Table S1 (page 5/5)**

| cpd # | Strucutural Group | Name | cpd ID | Structure | $K_I$ (µM) | $n_H$ |
|---|---|---|---|---|---|---|
| 23 | Others | N~1~-(4-bromo-3-methylphenyl)-N~3~-ethyl-N~3~-(2-methyl-2-propen-1-yl)-beta-alaninamide hydrochloride | 6507657 | 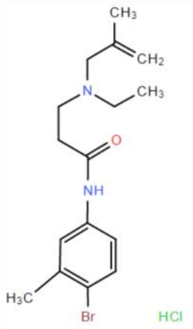 | 4 ± 0.3 | 1.4 |
| 41 | | 1-(4-chlorophenoxy)-3-(2,6-dimethyl-1-piperidinyl)-2-propanol hydrochloride | 5570600 | 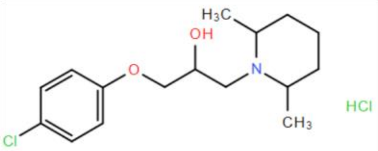 | 3 ± 0.3 | 1.8 |
| 35 | | (4-{[4-(4-fluorophenyl)-3,6-dihydro-1(2H)-pyridinyl]methyl}phenyl)dimethylamine | 5418213 | 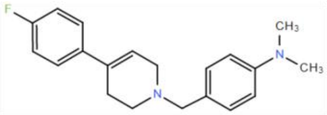 | 8 ± 0.7 | 1.1 |
| 42 | | N-(4-methoxyphenyl)-4-methyl-2-quinolinamine | 5569924 | 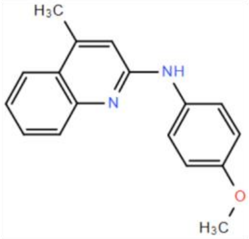 | 11 ± 2 | 0.6 |
| 11 | | 4-chloro-N-[3-(trifluoromethyl)phenyl]benzamide | 5259233 | 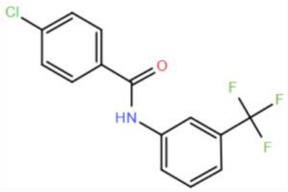 | 36 ± 2 | 2.4 |

**Table S1** – Compounds grouped according to structural similarities. $K_I$ ± standard error and $n_H$ for each compound were extracted from Hill equation fits to dose-response curves. Compound #24 was tested for dose-response but a $K_I$ could not be extracted.

**Table S2**

| Compounds that allowed a valinomycin response at the tested concentrations | | Compounds that did not allow full valinomycin response | |
|---|---|---|---|
| 36 | 18 | 31 (16 µM) | 32 (64 µM) |
| 13 | 41 | 20 (128 µM) | 23 (128 µM) |
| 45 | 42 | 37 (64 µM) | 15 (128 µM) |
| 26 | 43 | 8 (128 µM) | 33 (64 µM) |
| 30 | 44 | 25 (32 µM) | 38 (128 µM) |
| 27 | 16 | 7 (128 µM) | 1 (32 µM) |
| 15 | 12 | 3 (64 µM) | 39 (128 µM) |
| 4 | 10 | 29 (64 µM) | 21 (16 µM) |
| 35 | 11 | 5 (32 µM) | |
| 9 | 19 | 14 (64 µM) | |
| 40 | | 28 (32 µM) | |
| 17 | | 34 (64 µM) | |

**Table S2** – List of compounds that allowed a valinomycin response of empty liposomes under all concentrations tested and compounds that did not allow a valinomycin response above a certain concentration (in brackets). See **Fig. S5** for fluorescence curve examples.
